# Trends in coagulase-negative staphylococci (CoNS), England, 2010–2021

**DOI:** 10.1099/acmi.0.000491.v3

**Published:** 2023-06-19

**Authors:** Karthik Paranthaman, Allegra Wilson, Neville Verlander, Graeme Rooney, Neil Macdonald, Olisaeloka Nsonwu, Russell Hope, Paul Fleming, James Hatcher, Enitan Ogundipe, Natasha Ratnaraja, Yu Wan, Bruno Pichon, Samantha J. Westrop, Colin S. Brown, Alicia Demirjian

**Affiliations:** ^1^​ U.K. Health Security Agency, London, UK; ^2^​ Homerton Healthcare NHS Foundation Trust, London, UK; ^3^​ Queen Mary University of London, London, UK; ^4^​ Great Ormond Street Hospital for Children, London, UK; ^5^​ Chelsea and Westminster NHS Foundation Trust, London, UK; ^6^​ Imperial College London, London, UK; ^7^​ University Hospitals Coventry and Warwickshire NHS Trust, Coventry, UK; ^8^​ NIHR Health Protection Research Unit in Healthcare Associated Infections and Antimicrobial Resistance, Imperial College London, London, UK; ^9^​ Evelina London Children’s Hospital, London, UK; ^10^​ King’s College London, London, UK

**Keywords:** antimicrobial resistance, coagulase-negative staphylococci, epidemiology, species distribution

## Abstract

**Objective.:**

To review the epidemiology of coagulase-negative staphylococci (CoNS) in England over the recent 12 year period.

**Methods.:**

Laboratory-confirmed CoNS reported from sterile sites in patients in England to the UK Health Security Agency (UKHSA) between 2010 and 2021 were extracted from the national laboratory database and analysed.

**Results.:**

Overall, 668 857 episodes of CoNS were reported. Unspeciated CoNS accounted for 56 % (374 228) of episodes, followed by *Staphylococcus epidermidis* (26 %; 174 050), *S. hominis* (6.5 %; 43 501) and *S. capitis* (3.9 %; 25 773). Unspeciated CoNS increased by 8.2 % (95 % CI, 7.1–9.3) annually between 2010 and 2016, then decreased annually by 6.4 % (95 % CI: −4.8 to −7.9) until 2021. Speciated CoNS increased by 47.6 % (95 % CI, 44.5–50.9) annually between 2010 and 2016 and increased annually by 8.9 % (95 % CI: 5.1 to 12.8) until 2021. Antimicrobial susceptibility profiles differed by species.

**Conclusions.:**

Reports of CoNS from normally sterile body sites in patients in England increased between 2010 and 2016 and remained stable between 2017 and 2021. There has been a striking improvement in species-level identification of CoNS in recent years. Monitoring trends in CoNS epidemiology is crucial for development of observational and clinical intervention studies on individual species.

## Data Summary

The data presented in this paper is derived from the second-generation surveillance system (SGSS), the national database for laboratory reports of infectious diseases and antimicrobial resistance in England. As this database contains personal data, there are robust access controls in accordance with the Data Protection Act 2018, General Data Protection Regulations (GDPR) and the Caldicott Guidelines. All data presented in summary form in this paper are included in the Supplementary Material. Due to the risk of deductive disclosure, the authors are unable to share the raw data. UKHSA operates a robust governance process for access to protected data, where it is lawful, ethical and safe to do so. Organizations looking to access UKHSA data for public health purposes can contact DataAccess@ukhsa.gov.uk to request an application pack. Further information can be found on the UKHSA website (https://www.gov.uk/government/publications/accessing-ukhsa-protected-data/accessing-ukhsa-protected-data)

## Introduction

Coagulase-negative staphylococci (CoNS) are characterized by the absence of a major virulence factor called staphylococcal coagulase, in contrast to coagulase-positive staphylococci such as *Staphylococcus aureus *. There is substantial heterogeneity in virulence, habitat and antimicrobial-susceptibility profiles of CoNS with over 45 different species recognized to date [1–3]. Increasing adoption of matrix-assisted laser desorption/ionization time-of-flight (MALDI-TOF) mass spectrometry in recent years has enabled species-level identification of CoNS to be done more quickly and precisely than before. Correct identification to species-level identification is fundamental to understanding the clinical significance of CoNS [4].

Staphylococci commonly reside on the skin and mucous membranes of humans and animals [5]. Advances in healthcare have created conditions favourable to opportunistic CoNS infections, predominantly in hospitalized, vulnerable patients or those with indwelling catheters or devices. CoNS are adept at forming biofilms on abiotic and biotic surfaces, facilitating introduction of CoNS from skin to sterile sites following insertion of medical devices [1–3]. Vulnerable patients, particularly premature and low birthweight infants, are the most affected by CoNS due to prolonged hospital stays and more frequent application of invasive procedures [6–8]. However, detection of CoNS in a microbiological sample may represent contamination of the specimen from a colonized surface as a result of poor sampling and testing procedures, physiological colonization of skin and mucous membranes or a clinically significant infection [1, 3, 4]. A complicating factor is that CoNS species typically demonstrate drug resistance to beta-lactam antibiotics, including meticillin, limiting antimicrobial treatment options [1, 2, 4, 9]. It is likely that the health and economic burden of CoNS are underestimated [2].

Robust surveillance is essential to understand the increasing clinical importance of CoNS [6]. National surveillance arrangements for CoNS vary across and within countries. In England, diagnostic laboratories are required by law to report all clinically significant isolations of CoNS from sterile sites and antimicrobial susceptibility test results to the national surveillance system [10]. However, which CoNS isolates are speciated and whether it constitutes clinically significant infection is determined by local expert opinion. To better inform epidemiological and clinical understanding of different species, this analysis describes the trends in CoNS isolated from normally sterile body sites and reported to the national surveillance system between 2010 and 2021 in England.

## Methods

### Laboratory reporting arrangements

In England, diagnostic laboratories undertake testing of clinically relevant samples. While individual laboratory protocols on testing criteria, test methods, speciation and antimicrobial susceptibility testing may vary, guidance has been published on minimum standards [11].

The UK Health Security Agency (UKHSA) is responsible for infectious disease surveillance and public health protection in England. Diagnostic laboratories are required to notify UKHSA of specified causative organisms in human samples with core information such as patient identifiers, organism details, specimen type and specimen date, within 7 days. Reporting detections of staphylococci in all clinically significant isolates from sterile sites (e.g. blood, cerebrospinal fluid, joint fluid) is a legal requirement for diagnostic laboratories; however, reporting of antibiotic susceptibility data is voluntary. Diagnostic laboratories report susceptibility test results as susceptible, intermediate or resistant to selected antibiotics.

### National laboratory database

Reports submitted by diagnostic laboratories in England are stored by UKHSA in a database called the second-generation surveillance system (SGSS). SGSS comprises two modules, a communicable disease reporting module (CDR; formerly CoSurv/LabBase2) and an antimicrobial resistance module (AMR; formerly AmSurv). The CDR module includes reports of all organisms that are required to be reported to UKHSA by law. The AMR module contains antibiograms for all antimicrobials tested from all clinical sources.

The CDR records were deduplicated based on the OPIE (organism-patient-illness-episode) principle [10]. In brief, an episode constitutes each positive organism in a patient in a defined period. If the interval between two occasions where an individual was infected by the same organism was shorter than the defined episode length, the earlier report was included as a single episode and the latter report disregarded. Individuals infected with two or more organisms at the same time were represented by two (or more) distinct episodes. If the interval was longer than the defined episode length, they were represented by two distinct episodes. For CoNS, the episode length was defined as a rolling window of 14 days.

SGSS was launched in mid-2015. In the LabBase 2 and AmSurv systems that predated SGSS, linkage between CDR and AMR data was done by deterministic or probabilistic linkage methods. AMR data were stored at a test level and were linked to CDR OPIE records using common identifiers. If multiple AMR test results for the same antimicrobial was reported within the same patient episode, the most severe result (resistant) for the antimicrobial for that episode was chosen. LabBase 2 was estimated to capture approximately 80 % of records and missing data was unevenly distributed throughout the country. AmSurv was estimated to have even less complete coverage.

### Statistical analysis

Data on reports of CoNS from sterile sites were extracted from SGSS for the period 1 January 2010 to 31 December 2021. Annual counts were aggregated for all reported CoNS and separately for speciated and unspeciated CoNS. For speciated CoNS, annual counts were aggregated per species. After checks on data quality and cleaning, patient ages were grouped into five categories (0–11 months, 1–4 years, 5–17 years, 18–44 years, 45–64 years and 65 years and above) in line with the distribution of cases and clinical relevance. Sex and age-group-specific incidence rates for 100000 population per year were calculated using population data for England from the UK Office for National Statistics (ONS). We used the Wilson score method to calculate confidence intervals of incidence rates. Two-sided *P* values of 0.05 or less provided evidence of statistical association.

To describe trends, we analysed the data with all CoNS cases reported to UKHSA, with sub-classification to speciated and unspeciated CoNS. In addition, we conducted trend analysis for individual speciated CoNS. Due to overdispersion, we used negative binomial regression to quantify the average annual percent change (AAPC) with confidence intervals and to identify the points in time when trends changed. To assess changes in trend over 12 years of annual data, we specified a minimum of 4 years for each segment thus allowing a maximum of one change in trend for each species with full set of data points. For species with fewer than 12 data points, we removed years with no data prior to the first reported case and specified the maximum number of segments with the same requirement of four minimum data points for each segment. In the negative binomial model, the aggregated annual count of cases was included as outcome variable with year as continuous predictor variable and log of population for each year as offset. In addition, we ran models with an interaction term between year and all possible combinations of segment as factor variable. Further detail on the method and rationale is provided in the Supplementary Material, see Tables S1 and S2, available with the online version of this article. After checking all possible models to describe the trend, the best model was chosen by minimum the Bayesian information criterion (BIC). If the best fitting model had no interaction variable, the exponent of estimate for year variable gave incidence rate ratios (IRR) indicating the mean change per year. If the best fitting model included interaction between year and segment variable, the slope for each segment was derived using emtrends function in emmeans R package. The slope for each segment indicated the mean annual change over that segment. For interpretation, IRRs were converted to AAPC by taking the exponent of the estimate, subtracting by one and multiplying by 100.

As some species had a small number of cases in each year, we repeated the trend analysis by binomial regression and confirmed that the results were similar. Findings of negative binomial regression are presented in the paper. Changes in trend over time are reported as ‘increase’ or ‘decrease’ if there was evidence of statistical association for the segment (*P*<0.05) and as ‘stable’ otherwise.

Trends in antimicrobial susceptibility was explored for nine antimicrobials: ciprofloxacin, fusidic acid, meticillin, gentamicin, rifampicin, erythromycin, clindamycin, teicoplanin and vancomycin, for all reported CoNS and the five most common CoNS species during the study period. In accordance with EUCAST guidance, antimicrobial susceptibility results reported as intermediate were reclassified as susceptible [12]. While breakpoint criteria may have changed over time, susceptibility data reported in this study includes classification as they were reported at the time. Redesignation of intermediates to sensitive affected less than 0.1 % of isolates. Susceptibility data are reported as the proportion of isolates resistant to specific antimicrobials among all isolates reported to UKHSA with susceptibility data.

To better understand the reasons for improved species-level identification, we conducted a rapid survey of diagnostic laboratories in England in July 2021. To assess changes in speciation and reporting, the number of diagnostic laboratories reporting each species by year was summarized. Further detail is provided in the Supplementary Material.

UKHSA has legal permission to collect and analyse data for surveillance of infectious diseases in England.

## Results

A total of 668 857 episodes of CoNS from normally sterile sites were reported, of which unspeciated CoNS accounted for 56 % (374 228) of reported cases. Among the speciated CoNS, *S. epidermidis* (26 %, 174 050) was the most common, followed by *S. hominis* (6.5 %, 43 501) and *S. capitis* (3.9 %, 25 773). Samples from blood constituted 77.2 % (516 212) of the isolates, with the remaining coming from normally sterile tissue such as bone, joint or cerebrospinal fluid.

**Fig. 1.**
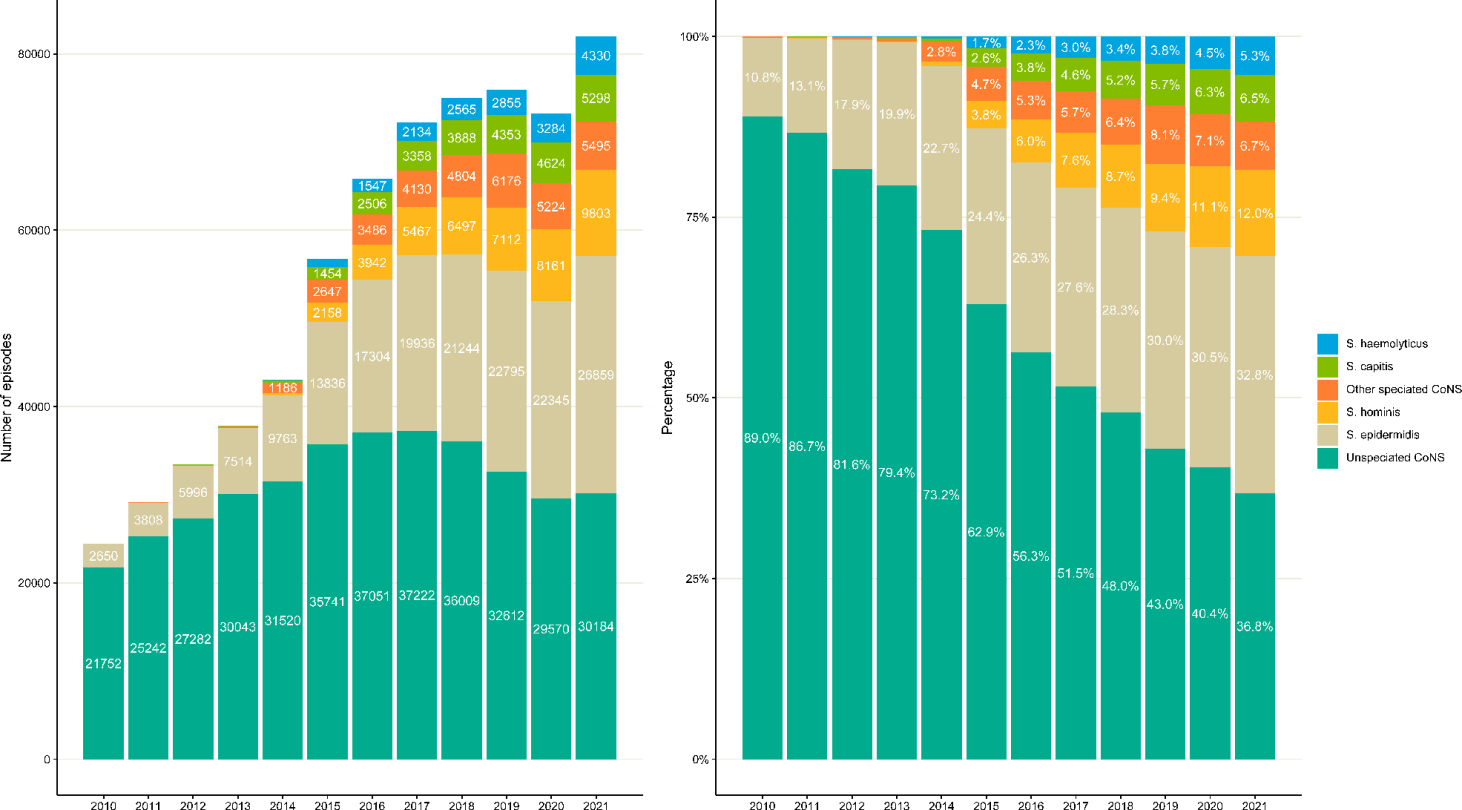
Annual counts and contribution of common CoNS species in sterile sites, England, 2015–2021.

Annual counts and contribution of common CoNS species are shown in Fig. 1 and Table S3. CoNS cases increased from 24 451 in 2010 to 81 969 cases in 2021. The proportion of unspeciated CoNS decreased from 89 % (95 % CI, 88.6–89.3, 21 752 cases) in 2010–36.8 % (95 % CI, 36.5–37.2 %, 30 184 cases) in 2021. In comparison, the proportion of speciated CoNS increased from 11% (95 % CI, 10.7–11.4 %, 2 699 cases) to 63.2 % (95 % CI, 62.8–63.5 %, 51 785 cases) in 2021. Annual incidence of CoNS increased from 46.4 (95 % CI, 45.9–47) per 100000 population in 2010 to 144.9 (95 % CI, 144.0 to 145.9) per 100 000 population in 2021. Annual incidence rates for CoNS by species are given in Tables S4 and S5.

**Fig. 2.**
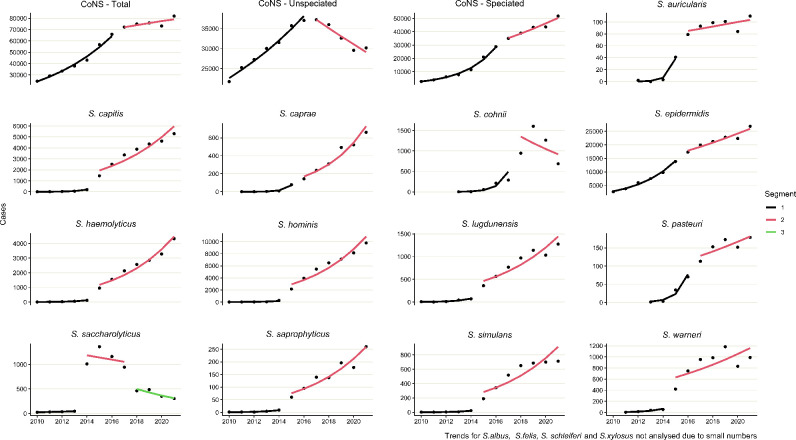
Counts and trend analysis of CoNS species in sterile sites, England, 2010–2021.

In the trend analysis (Tables 1 and 2, Fig. 2, Table S6), all CoNS increased annually by 16.8 % (95 % CI, 15.3 to 18.3) between 2010 and 2016 and were stable between 2017 and 2021 (AAPC 1.9 %; 95 % CI, −0.3 to 4.1). For the corresponding periods, speciated CoNS increased by 47.6 % (95 % CI, 44.4 to 50.9) and 8.9 % (95 % CI, 5.1 to 12.8), respectively. In contrast, unspeciated CoNS increased by 8.2 % (95 % CI, 7.1 to 9.3) between 2010 and 2016 and decreased by 6.4 % (95 % CI, −7.9 to −4.8) between 2017 and 2021.

**Table 1.** Trend analysis for CoNS, England, 2010–2021

Species	Period	Years	Annual percent change (95 % CI)	Trend
**All CoNS**	1	2010–2016	16.8 (15.3 to 18.3)	Increase
	2	2017–2021	1.9 (−0.3 to 4.1)	Stable
**Speciated CoNS**	1	2010–2016	47.6 (44.5 to 50.9)	Increase
	2	2017–2021	8.9 (5.1 to 12.8)	Increase
**Unspeciated CoNS**	1	2010–2016	8.2 (7.1 to 9.3)	Increase
	2	2017–2021	−6.4 (−7.9 to −4.8)	Decrease

**Table 2.** Trend analysis for CoNS species, England, 2010–2021

Species	Period	Years	Annual percent change (95 % CI)	Trend
*** S. auricularis ***	1	2012–2015	498.1 (225.7 to 998.4)	Increase
	2	2016–2021	3.5 (−1.4 to 8.7)	Stable
*** S. capitis ***	1	2010–2014	194.9 (143.5 to 257.2)	Increase
	2	2015–2021	20 (13.9 to 26.3)	Increase
*** S. caprae ***	1	2011–2015	365.6 (218.5 to 580.8)	Increase
	2	2016–2021	33.3 (26.5 to 40.5)	Increase
*** S. cohnii ***	1	2013–2017	242.5 (159.1 to 352.7)	Increase
	2	2018–2021	−12.3 (−35.1 to 18.4)	Stable
*** S. epidermidis ***	1	2010–2015	36.9 (33.8 to 40.2)	Increase
	2	2016–2021	7.3 (4.8 to 9.7)	Increase
*** S. haemolyticus ***	1	2010–2014	152.6 (111.1 to 202.4)	Increase
	2	2015–2021	24.3 (19.6 to 29.2)	Increase
*** S. hominis ***	1	2010–2014	107.3 (64.5 to 161.3)	Increase
	2	2015–2021	23.8 (10.5 to 38.7)	Increase
*** S. lugdunensis ***	1	2010–2014	142 (93.2 to 203)	Increase
	2	2015–2021	20.3 (13.8 to 27.3)	Increase
*** S. pasteuri ***	1	2013–2016	235.2 (152.1 to 345.8)	Increase
	2	2017–2021	8.6 (2 to 15.7)	Increase
*** S. saccharolyticus ***	1	2010–2013	18.5 (−0.3 to 40.8)	Stable
	2	2014–2017	−4.6 (−12.9 to 4.4)	Stable
	3	2018–2021	−14.9(−22.8 to −6.1)	Decrease
*** S. saprophyticus ***	1	2010–2014	102.5 (26.9 to 223)	Increase
	2	2015–2021	22.2 (16.4 to 28.3)	Increase
*** S. sciuri ***	1	2013–2021	65.9 (47.5 to 88)	Increase
*** S. simulans ***	1	2010–2014	146.3 (64.7 to 268.5)	Increase
	2	2015–2021	21.1 (12.3 to 30.5)	Increase
*** S. warneri ***	1	2011–2014	115.8 (61.6 to 188.1)	Increase
	2	2015–2021	10.1 (1.9 to 19)	Increase

Note: *S. albus, S. felis, S. schleiferi* and *S. xylosus* not analysed for trends due to small number of cases. *S. sciuri* has recently been designated as *Mammaliicoccus sciuri* and is no longer considered as belonging to CoNS group.

**Fig. 3.**
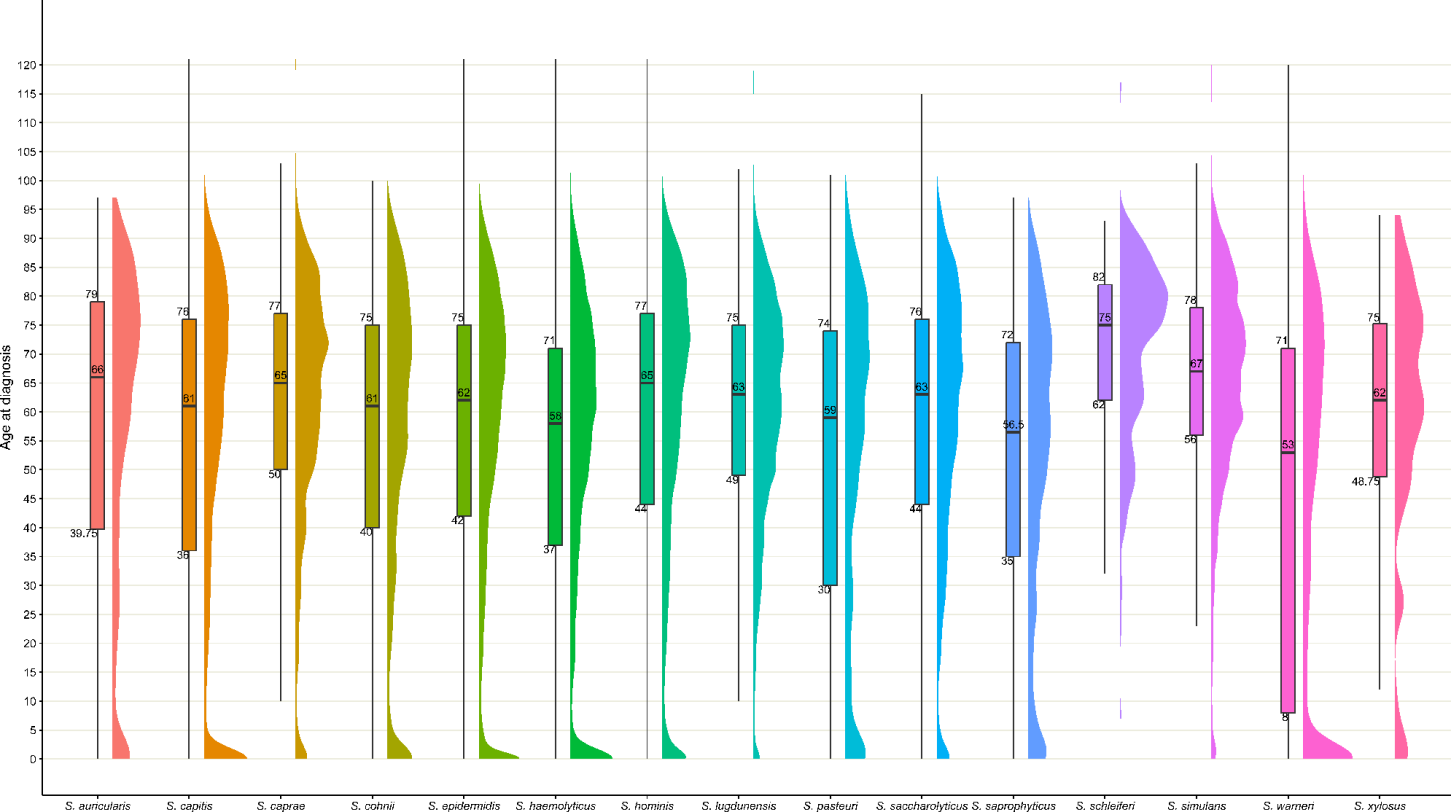
Age distribution of patients with CoNS species in sterile sites, England, 2010–2021.

The age at diagnosis showed a bimodal distribution, with peaks in early childhood and in the elderly (Fig. 3) for all CoNS species, except for *S. felis *, which had no cases reported in children up to 5 years of age.

**Fig. 4.**
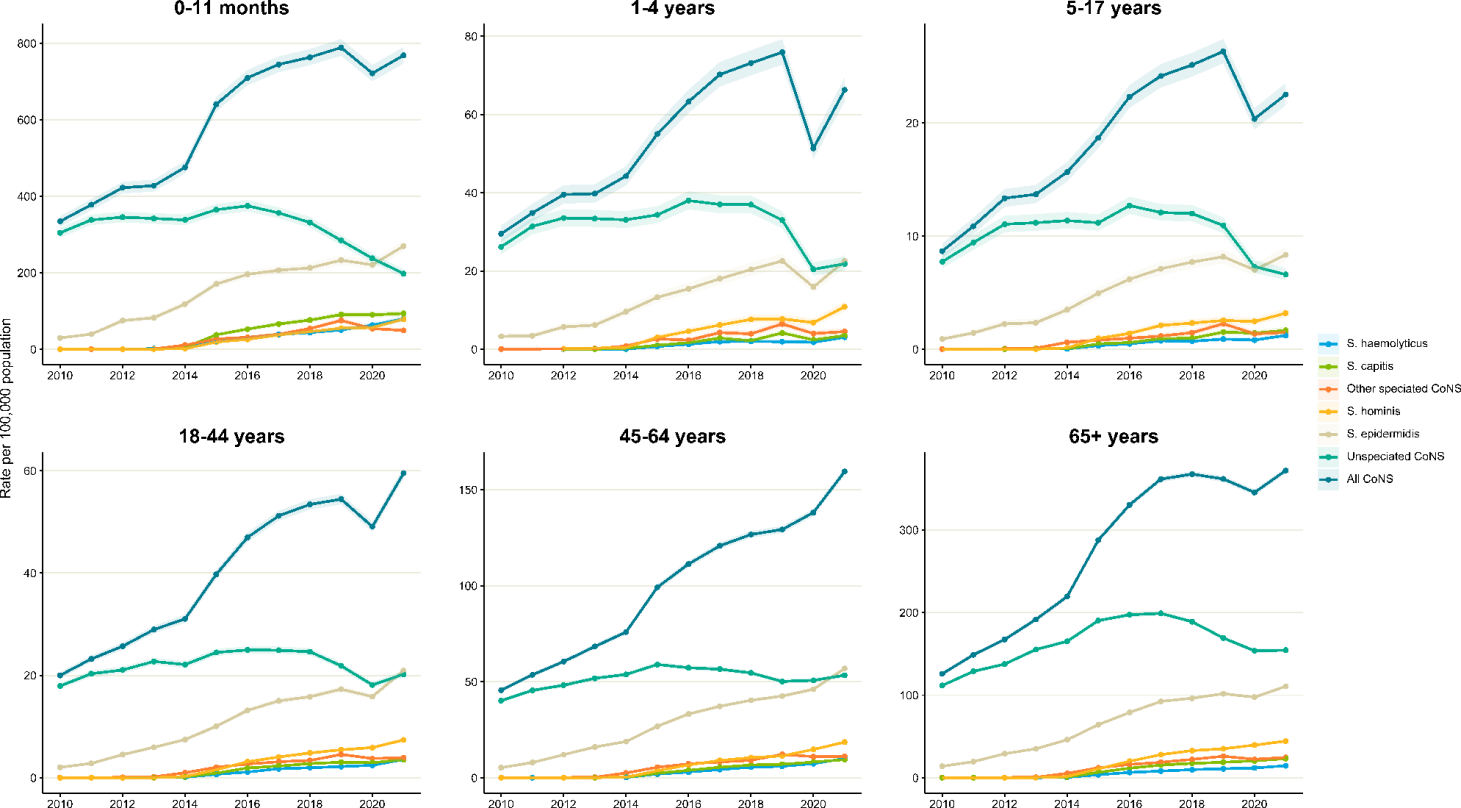
Annual incidence rate of common CoNS species by age group, England, 2010–2021.

Adults over the age of 65 years accounted for 48.5 % (324 454) of reported cases, followed by 45–64 year olds and 18–44 year olds at 25.0 % (167 608) and 14.1 % (94 285), respectively. Infants under 12 months of age accounted for 6.9 % (46 392) of reported cases. However, this age group had a higher incidence rate than all other age groups with rates increasing from 334.3 per 100 000 population in 2010 to 768.5 per 100 000 population in 2021 (Fig. 4). Adults aged 65 and above had the second highest incidence rates of 125.8 per 100 000 in 2010 increasing to 371.8 per 100 000 in 2021. Incidence rates for all CoNS in each age group is shown in Tables S7 and S8. Age distribution by CoNS species is shown in Table S9. Among infants, 60.1 % (27 888), 13.3 % (6195) and 7.1 % (3 326) of cases occurred in the first, second and third month after birth with the remaining 19.4 % (8 983) reported from 4 to 12 months of age. The contribution of common CoNS species is shown in Fig. 4 and age-group-specific incidence rates in Fig. S1. Unspeciated CoNS followed by *S. epidermidis* were most common among all age groups. *S. capitis* was the third most common CoNS species in infants, especially in the first 3 months of life. In all other age groups, *S. hominis* was the third most common CoNS species.

Overall incidence was higher for males (112.9 per 100000 population, 95 % CI, 112.5 to 113.2) than females (90.3 per 100000 population, 95 % CI, 89.9 to 90.6). The incidence rate ratio for males was 1.25(95 % CI, 1.24–1.26; *P*<0.001) compared to females overall and across all age groups (Fig. S2 and Table S10). Sex at birth was not recorded for 939 records (<0.01 %).

**Fig. 5.**
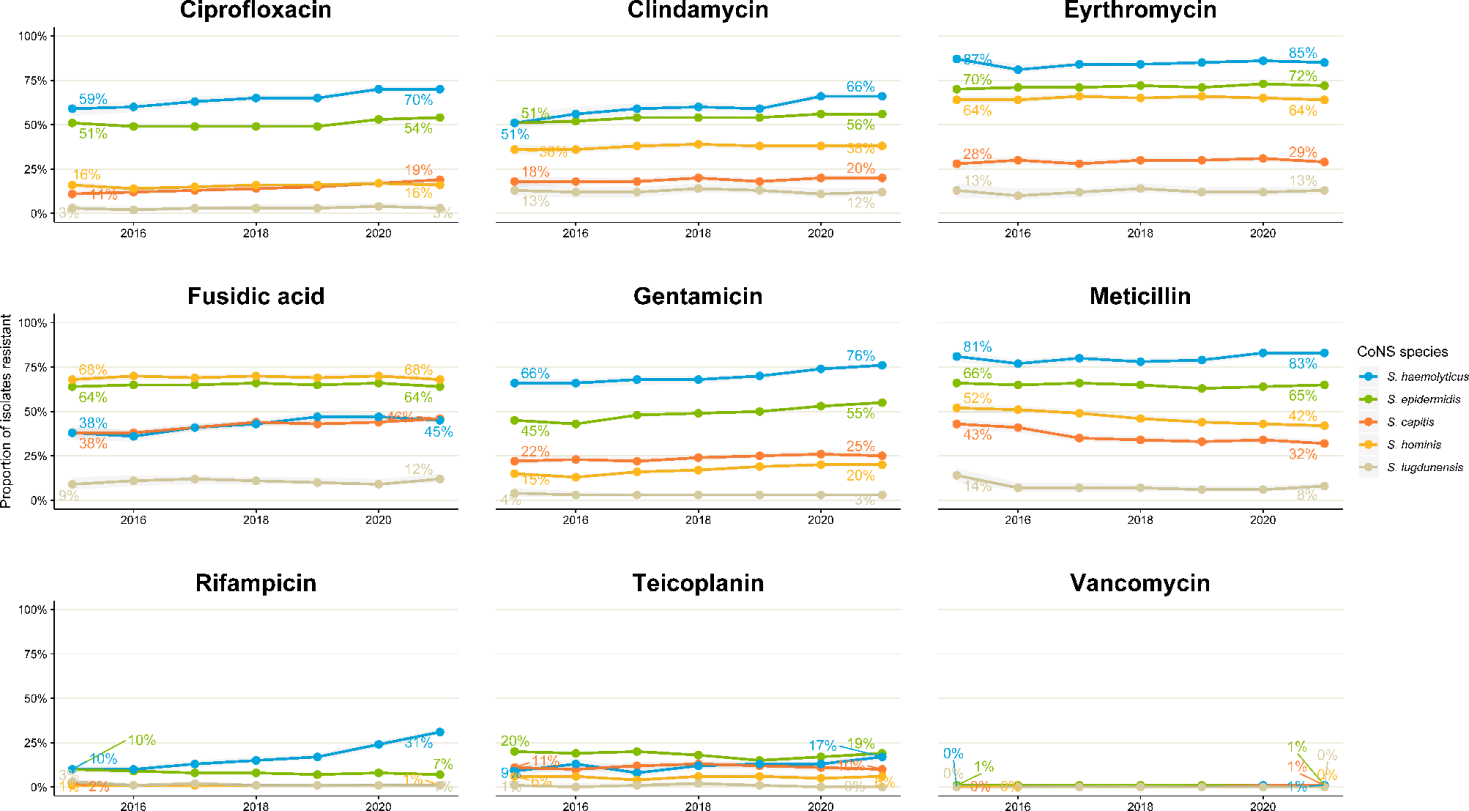
Trends in antimicrobial susceptibility for nine antimicrobials for CoNS in sterile sites, England, 2010–2021.

Trends in susceptibility to nine antibiotics for the five common CoNS species from 2015 and 2021 are presented in Fig. 5. The number of isolates with susceptibility data is shown in Fig. S3. Availability of information on susceptibility increased over time for all species except for *S. saccharolyticus *. Among all CoNS isolates, resistance trends were stable over time. Over 50 % of isolates were resistant to erythromycin, fusidic acid and meticillin, between 25 and 50 % were reported as resistant to clindamycin, ciprofloxacin and gentamicin and less than 25 % for teicoplanin, vancomycin and rifampicin. There were marked differences in antimicrobial susceptibility by species. *S. capitis* and *S. haemolyticus* showed lower and higher levels of resistance for the nine antibiotics, respectively, compared to overall resistance trends of CoNS. Further details of the proportions of resistant isolates for all CoNS and the five most common species is given in Tables S10–S16.

Excluding unspeciated CoNS, the median number of unique speciated CoNS reported by diagnostic laboratories increased from one in 2010, to four (Interquartile range[IQR], 2 to 8) in 2015 to nine in 2021 (IQR, 6 to 12). Additional information on the number of laboratories reporting different CoNS species by year is given in Figs S4 and S5.

The rapid laboratory survey found that among 75 laboratories that were able to speciate CoNS, 61 reported using MALDI-TOF and the rest used other methods such as analytical profile index (API) and Vitek 2 for speciation. Over half of laboratories surveyed (57 % *n*=43) reported that they speciated CoNS detected from sterile sites only, whereas 15 % (*n*=11) reported speciating CoNS detected from all sites and the rest reported using other criteria for speciation.

## Discussion

The overall rate of CoNS increased between 2010 and 2016 and stabilized between 2017 and 2021. The increase in CoNS predated the improvements in laboratory reporting procedures in England in 2014–15, with the year 2016 representing the first full year of strengthened mandatory reporting. Identification of CoNS to species-level increased substantially in the last 6 years, with important implications for epidemiological surveillance and antibiotic treatment recommendations.

Infants under 12 months of age have the highest rates for CoNS and were especially at higher risk in the first month of life. This likely reflects medical device-related infections in preterm newborns, and previous studies have shown that biofilm-producing staphylococci are associated with colonization and infection among preterm neonates [13]. Adults aged over 65 years of age also had high rates of CoNS, most likely linked to multimorbidity, waning immunity and/or immunosuppressive treatment. We also detected a higher risk in males compared to females across all age groups.

Some changes relating to classification of CoNS in recent years must be noted. In 2020, *S. sciuri* was moved out of *Staphylococcus* to the novel genus *Mammaliicoccus* and renamed as *Mammaliicoccus sciuri *. Additionally, *S. schleiferi* reported in this study could have contained the subspecies *S. schleiferi coagulans,* which has been redesignated as *S. coagulans *. [14]. Given these changes were made recently, there were no reports of *S. coagulans* in the study period in England.

We noted high levels (55–85 %) of antimicrobial resistance among CoNS, especially for *S. haemolyticus* and *S. epidermidis* against erythromycin, gentamicin and methicillin. While there are some differences, the antimicrobial resistance patterns found in this study are broadly consistent with a recent study from the Netherlands [15]. Compared to other CoNS species, *S. lugdunensis* was susceptible to most antibiotics and this finding is consistent with the current literature [16]. Monitoring and reviewing AMR trends by age group and site of specimen would be critical in providing appropriate antimicrobial recommendations once species-level identification is made.

There are several limitations to this study. First, as the laboratory reporting database did not contain clinical data, we could not distinguish whether the reported detection of CoNS represented a clinically relevant infection or contamination of specimen during sampling and processing. There remains considerable uncertainty on the interpretation of CoNS in sterile sites and there are currently no single objective markers that could be used systematically to interpret national surveillance data [1]. Second, reporting of CoNS was unlikely to be consistent during the study period. In particular, laboratory protocols for testing, detection and identification of CoNS may vary over time. Local variation may also play a role, in particular, local microbiology expertise was cited by a number of laboratories as criteria for whether or not an isolate was speciated, antimicrobial susceptibility testing was performed, and/or results were reported to requesting clinicians. As noted, there were also improvements in laboratory reporting to UKHSA around 2015. Third, although almost all laboratories reported the ability to speciate CoNS, and improvements have been made in recent years, there is some evidence to suggest that under-reporting of certain CoNS species to the national surveillance system is still occurring. Finally, data for 2020 may not be representative of secular trends in view of the disruption to routine healthcare services by the COVID-19 pandemic.

Nevertheless, this retrospective cross-sectional study of CoNS detected in sterile sites, linking it with AMR data over the 12 year period provides real-world data on epidemiology of CoNS in England. The high burden of CoNS highlights the central role of infection control measures in minimizing transmission and severe outcomes related to healthcare. Improving speciation methods for CoNS offers scope for more in-depth studies on clinical importance, risk factors and outcomes at species level. Infants under 12 months of age have the highest incidence rates and a substantial proportion of this burden occurs within the first 3 months after birth. Monitoring trends in CoNS epidemiology will allow for more real-time development of observational and clinical intervention studies. Further studies linking laboratory data with clinical and outcome data will allow for more informed decisions on clinical management and more targeted surveillance and intervention measures.

## Supplementary Data

Supplementary material 1
