# Supplementary material for: Trends in coagulase-negative staphylococci (CoNS), England, 2010–2021
Source: Access Microbiol. 2023 Jun 19;5(6):acmi000491.v3. doi: 10.1099/acmi.0.000491.v3 (PMC10323795; doi:10.1099/acmi.0.000491.v3)

Supplementary Appendix

Supplement to: Trends in Coagulase-negative Staphylococci, England, 2010-2021

This appendix has been provided by the authors to give readers additional information about the work.

Table of contents

1. Supplementary methods 3

List of tables

Table S1: Possible models for trends analysis of 12 years 4
Table S2: Possible models for trends analysis of 10 years 4
Table S3: Counts and proportion of CoNS species 5
Table S4: Annual rates (per 100,000) of CoNS 6
Table S5: Annual rates (per 100,000) of CoNS species 6
Table S6: Trend analysis of CoNS species 7
Table S7: Annual incidence rate of common CoNS species by age group 10
Table S8: Annual incidence rates of CoNS by age group 13
Table S9: Age distribution (in years) of CoNS species 16
Table S10: Annual incidence rates (per 100,000) by sex and age group 17
Table S11: Proportion of isolates resistant to antimicrobials - all CoNS 20
Table S12: Proportion of isolates resistant to antimicrobials - *S. epidermidis* 21
Table S13: Proportion of isolates resistant to antimicrobials - *S. capitis* 22
Table S14: Proportion of isolates resistant to antimicrobials - *S. haemolyticus* 23
Table S15: Proportion of isolates resistant to antimicrobials - *S. hominis* 24
Table S16: Proportion of isolates resistant to antimicrobials - *S. lugdunensis* 25

List of figures

Figure S1. Contribution of common CoNS species by age group 14
Figure S2. Annual incidence rates by sex and age group 15
Figure S3. Number of isolates with susceptibility data 19
Figure S4. Number of laboratories reporting yearly by CoNS species 26
Figure S5. Number of unique species reported by laboratories by year 27

1. Supplementary methods

Generalised linear models with negative binomial distribution were used to analyse trends. Aggregated annual count of cases for each group/species was specified as outcome variable, year as continuous explanatory variable and log of the population for year as offset were included in all models.

To assess changes in trends, we performed all possible models specifying at least four years for each segment. Given 12 years of data, the maximum number of segments was three. The minimum length of segment was chosen as a pragmatic approach to describe trends, given the restriction of 12 years of annual counts. For illustration, all possible models for species with 12 years and 10 years of data are shown below.

The cell values in Tables 1 and 2 for Model columns indicate the segment specification for each year. In Table S1 with 12 years of data, Model 1 is the base model with year included as continuous variable and no segments. Model 1 assumes constant change over time and has no segments. Model 2 includes two segments with the first segment from years 1 to 7 and second segment from year 8 and 11, satisfying the minimum four years condition for each segment. In Model 2, explanatory variables are year as continuous variable, segment as factor variable and an interaction term for year and segment variable. Models 3 to 7 are other possible combinations satisfying the condition of minimum four years for each segment. In Table S2 with 10 years of data, four models are possible. In both scenarios, no other models are possible with the condition of four years for each segment.

After running all possible models for each species, the model with minimum BIC value was chosen as the best model.

Table S1: Possible models for trends analysis of 12 years

Year	Segment						
	Model 1	Model 2	Model 3	Model 4	Model 5	Model 6	Model 7
1	1	1	1	1	1	1	1
2	1	1	1	1	1	1	1
3	1	1	1	1	1	1	1
4	1	1	1	1	1	1	1
5	1	1	1	1	1	2	2
6	1	1	1	1	2	2	2
7	1	1	1	2	2	2	2
8	1	1	2	2	2	2	2
9	1	2	2	2	2	2	3
10	1	2	2	2	2	2	3
11	1	2	2	2	2	2	3
12	1	2	2	2	2	2	3

Table S2: Possible models for trends analysis of 10 years

Year	Segment			
	Model 1	Model 2	Model 3	Model 4
1	1	1	1	1
2	1	1	1	1
3	1	1	1	1
4	1	1	1	1
5	1	1	1	2
6	1	1	2	2
7	1	2	2	2
8	1	2	2	2
9	1	2	2	2
10	1	2	2	2

Table S3: Counts and proportion of CoNS species

Species	2010	2011	2012	2013	2014	2015	2016	2017	2018	2019	2020	2021
**Unspeciated CoNS**	21752 (89.0%)	25242 (86.7%)	27282 (81.6%)	30043 (79.4%)	31520 (73.2%)	35741 (62.9%)	37051 (56.3%)	37222 (51.5%)	36009 (48.0%)	32612 (43.0%)	29570 (40.4%)	30184 (36.8%)
S. epidermidis	2650 (10.8%)	3808 (13.1%)	5996 (17.9%)	7514 (19.9%)	9763 (22.7%)	13836 (24.4%)	17304 (26.3%)	19936 (27.6%)	21244 (28.3%)	22795 (30.0%)	22345 (30.5%)	26859 (32.8%)
S. haemolyticus	1 (0.0%)	6 (0.0%)	24 (0.1%)	50 (0.1%)	113 (0.3%)	952 (1.7%)	1547 (2.3%)	2134 (3.0%)	2565 (3.4%)	2855 (3.8%)	3284 (4.5%)	4330 (5.3%)
S. hominis	12 (0.0%)	25 (0.1%)	23 (0.1%)	33 (0.1%)	268 (0.6%)	2158 (3.8%)	3942 (6.0%)	5467 (7.6%)	6497 (8.7%)	7112 (9.4%)	8161 (11.1%)	9803 (12.0%)
S. capitis	1 (0.0%)	6 (0.0%)	32 (0.1%)	57 (0.2%)	196 (0.5%)	1454 (2.6%)	2506 (3.8%)	3358 (4.6%)	3888 (5.2%)	4353 (5.7%)	4624 (6.3%)	5298 (6.5%)
**Other speciated CoNS**	35 (0.1%)	40 (0.1%)	71 (0.2%)	150 (0.4%)	1186 (2.8%)	2647 (4.7%)	3486 (5.3%)	4130 (5.7%)	4804 (6.4%)	6176 (8.1%)	5224 (7.1%)	5495 (6.7%)

Table S4: Annual rates (per 100,000) of CoNS

Species	2010	2011	2012	2013	2014	2015	2016	2017	2018	2019	2020	2021
**All CoNS**	46.4	54.8	62.5	70.3	79.3	103.7	119.1	129.9	134.0	134.9	129.5	144.9
**Speciated CoNS**	5.1	7.3	11.5	14.5	21.2	38.4	52.1	63.0	69.7	76.9	77.2	91.6
**Unspeciated CoNS**	41.3	47.5	51.0	55.8	58.0	65.2	67.0	66.9	64.3	57.9	52.3	53.4

Table S5: Annual rates (per 100,000) of CoNS species

Species	2010	2011	2012	2013	2014	2015	2016	2017	2018	2019	2020	2021
S. auricularis						0.1	0.1	0.2	0.2	0.2	0.1	0.2
S. capitis			0.1	0.1	0.4	2.7	4.5	6.0	6.9	7.7	8.2	9.4
S. caprae						0.1	0.3	0.4	0.6	0.9	0.9	1.2
S. cohnii						0.1	0.4	0.5	1.7	2.8	2.2	1.2
S. epidermidis	5.0	7.2	11.2	13.9	18.0	25.3	31.3	35.8	38.0	40.5	39.5	47.5
S. haemolyticus				0.1	0.2	1.7	2.8	3.8	4.6	5.1	5.8	7.7
S. hominis				0.1	0.5	3.9	7.1	9.8	11.6	12.6	14.4	17.3
S. lugdunensis				0.1	0.1	0.7	1.0	1.4	1.7	2.0	1.8	2.3
S. pasteuri						0.1	0.1	0.2	0.3	0.3	0.3	0.3
S. saccharolyticus	0.1	0.1	0.1	0.1	1.9	2.5	2.1	1.7	0.8	0.9	0.6	0.5
S. saprophyticus						0.1	0.2	0.2	0.2	0.3	0.3	0.5
S. simulans						0.3	0.6	0.9	1.2	1.2	1.2	1.3
S. warneri				0.1	0.1	0.8	1.4	1.7	1.8	2.1	1.5	1.7

Note: *S.albus*, *S.felis*, *S.schleiferi* and *S. xylosus* not shown due to small number of cases

Table S6: Trend analysis of CoNS species

Species	Variable	2010	2011	2012	2013	2014	2015	2016	2017	2018	2019	2020	2021
**CoNS - Total**	Observed cases	24451	29127	33428	37847	43046	56788	65836	72247	75007	75903	73208	81969
	Modelled cases	24117	28411	33418	39296	46272	54501	64204	72095	73934	75752	77548	79018
	Segment	1	1	1	1	1	1	1	2	2	2	2	2
**CoNS - Unspeciated**	Observed cases	21752	25242	27282	30043	31520	35741	37051	37222	36009	32612	29570	30184
	Modelled cases	22621	24696	26921	29336	32012	34943	38147	37247	35100	33048	31089	29111
	Segment	1	1	1	1	1	1	1	2	2	2	2	2
**CoNS - Speciated**	Observed cases	2699	3885	6146	7804	11526	21047	28785	35025	38998	43291	43638	51785
	Modelled cases	2618	3900	5800	8622	12836	19115	28469	35219	38604	42276	46258	50379
	Segment	1	1	1	1	1	1	1	2	2	2	2	2
S. auricularis	Observed cases			<10	<10	<10	41	79	93	99	101	84	110
	Modelled cases			0	1	6	38	85	89	92	96	100	104
	Segment			1	1	1	1	2	2	2	2	2	2
S. capitis	Observed cases	<10	<10	32	57	196	1454	2506	3358	3888	4353	4624	5298
	Modelled cases	3	7	22	66	196	1943	2352	2839	3427	4134	4982	5976
	Segment	1	1	1	1	1	2	2	2	2	2	2	2
S. caprae	Observed cases		<10	<10	<10	<10	79	144	237	311	493	522	661
	Modelled cases		0	1	3	15	73	169	227	305	408	547	728

Species	Variable	2010	2011	2012	2013	2014	2015	2016	2017	2018	2019	2020	2021
	Segment		1	1	1	1	1	2	2	2	2	2	2
S. cohnii	Observed cases				<10	<10	56	211	288	946	1601	1262	686
	Modelled cases				3	12	41	143	493	1349	1189	1047	918
	Segment				1	1	1	1	1	2	2	2	2
S. epidermidis	Observed cases	2650	3808	5996	7514	9763	13836	17304	19936	21244	22795	22345	26859
	Modelled cases	2813	3885	5358	7388	10200	14087	17861	19277	20807	22438	24177	25929
	Segment	1	1	1	1	1	1	2	2	2	2	2	2
S. haemolyticus	Observed cases	<10	<10	24	50	113	952	1547	2134	2565	2855	3284	4330
	Modelled cases	3	7	19	47	120	1174	1473	1843	2307	2884	3603	4481
	Segment	1	1	1	1	1	2	2	2	2	2	2	2
S. hominis	Observed cases	12	25	23	33	268	2158	3942	5467	6497	7112	8161	9803
	Modelled cases	9	19	40	83	173	2927	3655	4553	5673	7061	8782	10871
	Segment	1	1	1	1	1	2	2	2	2	2	2	2
S. lugdunensis	Observed cases	<10	<10	11	41	69	360	563	765	971	1140	1032	1276
	Modelled cases	2	5	13	31	76	461	559	677	820	992	1199	1443
	Segment	1	1	1	1	1	2	2	2	2	2	2	2
S. pasteuri	Observed cases				<10	<10	34	70	113	153	173	152	179
	Modelled cases				2	7	23	77	128	140	153	167	181

Species	Variable	2010	2011	2012	2013	2014	2015	2016	2017	2018	2019	2020	2021
	Segment				1	1	1	1	2	2	2	2	2
S. saccharolyticus	Observed cases	27	33	36	47	1009	1359	1161	942	463	485	352	305
	Modelled cases	27	32	38	46	1184	1139	1096	1052	500	428	366	312
	Segment	1	1	1	1	2	2	2	2	3	3	3	3
S. saprophyticus	Observed cases	<10	<10	<10	<10	<10	60	95	139	138	196	178	260
	Modelled cases	0	1	2	4	8	76	93	115	141	173	212	259
	Segment	1	1	1	1	1	2	2	2	2	2	2	2
S. simulans	Observed cases	<10	<10	<10	<10	23	189	342	519	651	687	700	712
	Modelled cases	1	1	3	8	21	281	344	419	510	621	755	914
	Segment	1	1	1	1	1	2	2	2	2	2	2	2
S. warneri	Observed cases		<10	15	40	54	422	747	955	986	1184	832	989
	Modelled cases		6	14	30	66	631	700	776	860	952	1053	1159
	Segment		1	1	1	1	2	2	2	2	2	2	2

Table S7: Annual incidence rate of common CoNS species by age group

Age group	Species	2010	2011	2012	2013	2014	2015	2016	2017	2018	2019	2020	2021
**0-11 months**	All CoNS	334.3	377.9	422.3	427.6	475.8	640.4	709.9	744.8	763.5	788.9	721.7	768.5
	Unspeciated CoNS	304.4	338.2	345.3	341.9	338.2	365.0	375.0	356.6	331.4	284.6	237.6	197.7
	S. epidermidis	29.8	39.5	74.8	82.3	118.2	170.4	195.8	206.6	212.8	232.8	220.6	269.8
	S. hominis	0.1		0.1	0.4	1.5	18.7	25.9	37.5	45.9	54.9	56.8	78.7
	S. capitis			0.6	0.6	4.7	37.7	52.6	66.1	76.2	90.7	89.7	93.5
	S. haemolyticus			0.7	1.3	2.6	21.9	29.4	38.9	43.4	50.6	63.0	79.6
	Other speciated CoNS		0.1	0.7	1.0	10.7	26.7	31.2	39.2	53.8	75.3	54.0	49.2
**1-4 years**	All CoNS	29.5	34.9	39.5	39.8	44.3	55.0	63.3	70.2	73.2	75.9	51.4	66.3
	Unspeciated CoNS	26.2	31.4	33.6	33.4	33.1	34.3	38.0	37.0	37.0	33.0	20.4	21.8
	S. epidermidis	3.3	3.4	5.7	6.2	9.6	13.3	15.4	18.0	20.4	22.6	15.9	22.6
	S. hominis			0.1		0.4	3.0	4.6	6.2	7.6	7.8	6.8	10.8
	S. capitis			0.1		0.3	1.0	1.7	2.8	2.2	4.2	2.4	3.5
	S. haemolyticus			0.1			0.7	1.3	1.9	2.0	1.9	1.8	3.1
	Other speciated CoNS				0.1	0.8	2.7	2.3	4.2	3.9	6.5	4.0	4.5
**5-17 years**	All CoNS	8.7	10.9	13.4	13.7	15.7	18.7	22.3	24.1	25.1	26.3	20.4	22.5
	Unspeciated CoNS	7.7	9.4	11.0	11.2	11.4	11.2	12.7	12.1	12.0	10.9	7.3	6.6

Age group	Species	2010	2011	2012	2013	2014	2015	2016	2017	2018	2019	2020	2021
	S. epidermidis	0.9	1.4	2.2	2.3	3.5	4.9	6.2	7.1	7.7	8.2	7.0	8.3
	S. hominis					0.1	1.0	1.4	2.1	2.3	2.5	2.5	3.2
	S. capitis						0.5	0.6	0.9	1.0	1.5	1.4	1.7
	S. haemolyticus						0.3	0.5	0.7	0.7	0.9	0.8	1.2
	Other speciated CoNS				0.1	0.6	0.8	1.0	1.2	1.5	2.3	1.4	1.5
**18-44 years**	All CoNS	20.0	23.2	25.7	29.0	31.1	39.8	47.0	51.2	53.4	54.4	49.1	59.5
	Unspeciated CoNS	17.9	20.3	21.1	22.7	22.1	24.5	25.0	24.9	24.6	21.9	18.1	20.2
	S. epidermidis	2.0	2.8	4.5	5.9	7.5	10.1	13.2	15.0	15.8	17.3	15.9	20.9
	S. hominis					0.2	1.6	3.1	4.1	4.8	5.5	5.9	7.4
	S. capitis					0.2	0.9	1.9	2.3	2.8	3.1	2.9	3.5
	S. haemolyticus					0.1	0.7	1.1	1.8	2.0	2.2	2.4	3.6
	Other speciated CoNS			0.1	0.2	1.0	2.1	2.7	3.1	3.4	4.6	3.8	3.9
**45-64 years**	All CoNS	45.6	53.6	60.5	68.3	75.9	99.2	111.3	120.8	126.7	129.2	138.1	159.4
	Unspeciated CoNS	40.2	45.5	48.2	51.8	53.8	59.0	57.3	56.5	54.6	50.2	50.7	53.4
	S. epidermidis	5.3	8.0	12.0	16.0	18.9	26.8	33.2	37.2	40.3	42.6	46.1	56.9
	S. hominis	0.1			0.1	0.3	3.6	6.7	9.0	10.5	11.4	14.8	18.6
	S. capitis			0.1	0.1	0.2	2.4	3.9	5.5	6.6	7.0	8.2	9.4

Age group	Species	2010	2011	2012	2013	2014	2015	2016	2017	2018	2019	2020	2021
	S. haemolyticus				0.1	0.2	1.9	3.0	4.3	5.5	6.0	7.4	10.0
	Other speciated CoNS	0.1	0.1	0.1	0.3	2.5	5.5	7.2	8.2	9.1	12.2	11.0	11.2
**65+ years**	All CoNS	125.8	148.9	167.5	191.6	219.3	287.7	330.3	361.5	367.6	361.7	345.4	371.8
	Unspeciated CoNS	111.7	128.8	137.6	155.3	165.0	190.3	197.3	199.0	188.9	169.2	153.6	154.5
	S. epidermidis	13.9	19.6	29.1	35.1	46.0	64.3	79.0	92.4	96.3	101.7	97.6	110.7
	S. hominis		0.2	0.1	0.2	1.5	10.9	20.0	28.0	32.9	35.1	39.5	44.3
	S. capitis			0.2	0.3	0.9	6.5	11.5	15.4	17.4	18.8	20.4	23.3
	S. haemolyticus			0.1	0.2	0.5	3.7	6.4	8.1	9.9	10.9	11.9	14.5
	Other speciated CoNS	0.2	0.2	0.3	0.6	5.3	12.0	16.1	18.7	22.2	26.1	22.4	24.6

Table S8: Annual incidence rates of CoNS by age group

Age group	2010	2011	2012	2013	2014	2015	2016	2017	2018	2019	2020	2021
**0-11 months**	334.3 (2247)	377.9 (2566)	422.3 (2941)	427.6 (2893)	475.8 (3160)	640.4 (4246)	709.9 (4750)	744.8 (4867)	763.5 (4870)	788.9 (4882)	721.7 (4344)	768.5 (4626)
**1-4 years**	29.5 (770)	34.9 (924)	39.5 (1066)	39.8 (1089)	44.3 (1225)	55 (1525)	63.3 (1747)	70.2 (1918)	73.2 (1982)	75.9 (2036)	51.4 (1355)	66.3 (1749)
**5-17 years**	8.7 (694)	10.9 (872)	13.4 (1072)	13.7 (1108)	15.7 (1278)	18.7 (1540)	22.3 (1865)	24.1 (2048)	25.1 (2163)	26.3 (2296)	20.4 (1803)	22.5 (1993)
**18-44 years**	20 (3904)	23.2 (4540)	25.7 (5012)	29 (5634)	31.1 (6042)	39.8 (7758)	47 (9176)	51.2 (9983)	53.4 (10420)	54.4 (10619)	49.1 (9577)	59.5 (11620)
**45-64 years**	45.6 (6061)	53.6 (7229)	60.5 (8171)	68.3 (9290)	75.9 (10422)	99.2 (13774)	111.3 (15653)	120.8 (17170)	126.7 (18156)	129.2 (18619)	138.1 (19987)	159.4 (23076)
**65+ years**	125.8 (10775)	148.9 (12996)	167.5 (15166)	191.6 (17833)	219.3 (20919)	287.7 (27945)	330.3 (32645)	361.5 (36261)	367.6 (37416)	361.7 (37451)	345.4 (36142)	371.8 (38905)

Note: Numbers in parenthesis indicate count of cases in each group

Figure S1. Contribution of common CoNS species by age group

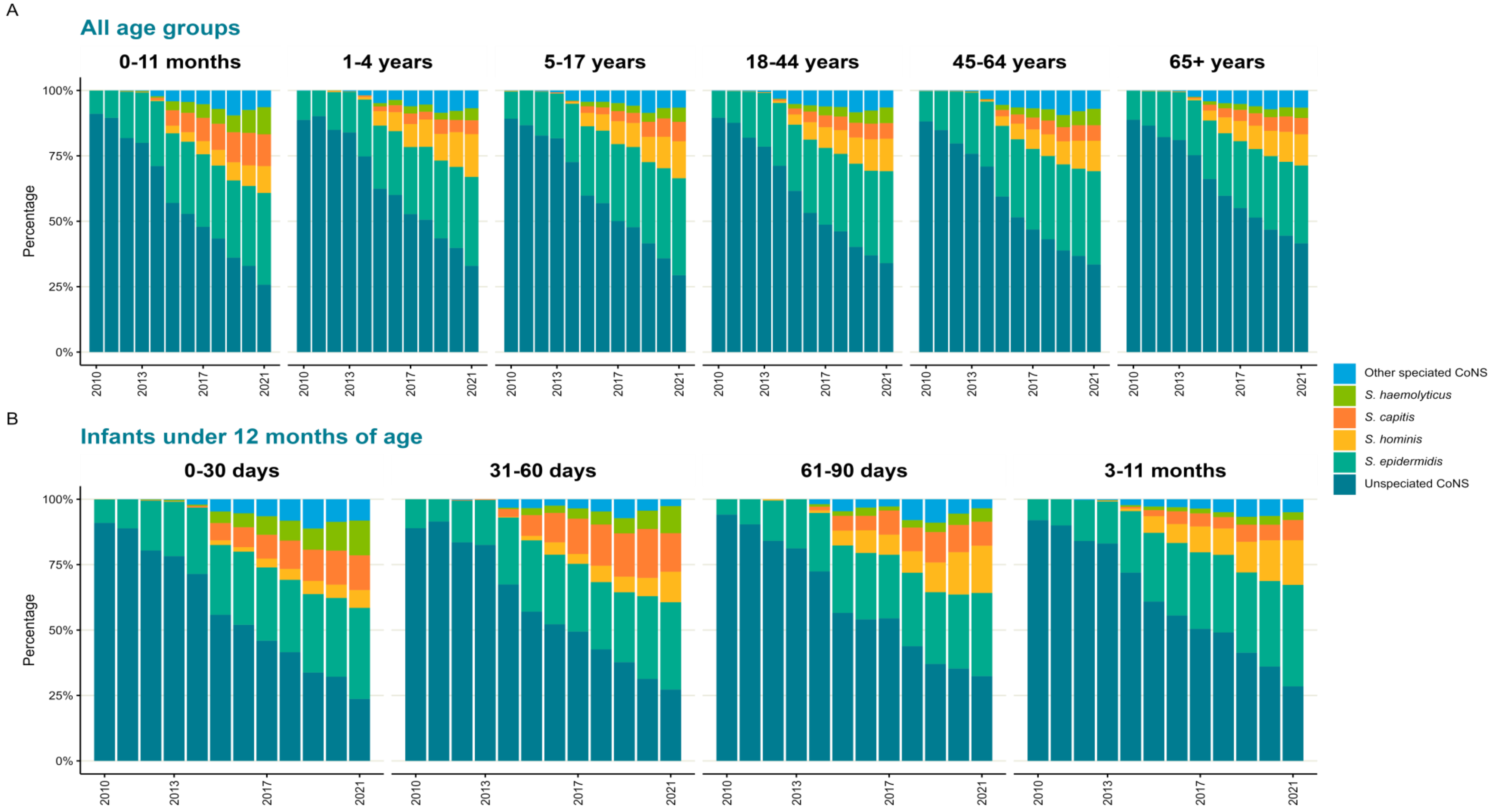

Figure S2. Annual incidence rates by sex and age group

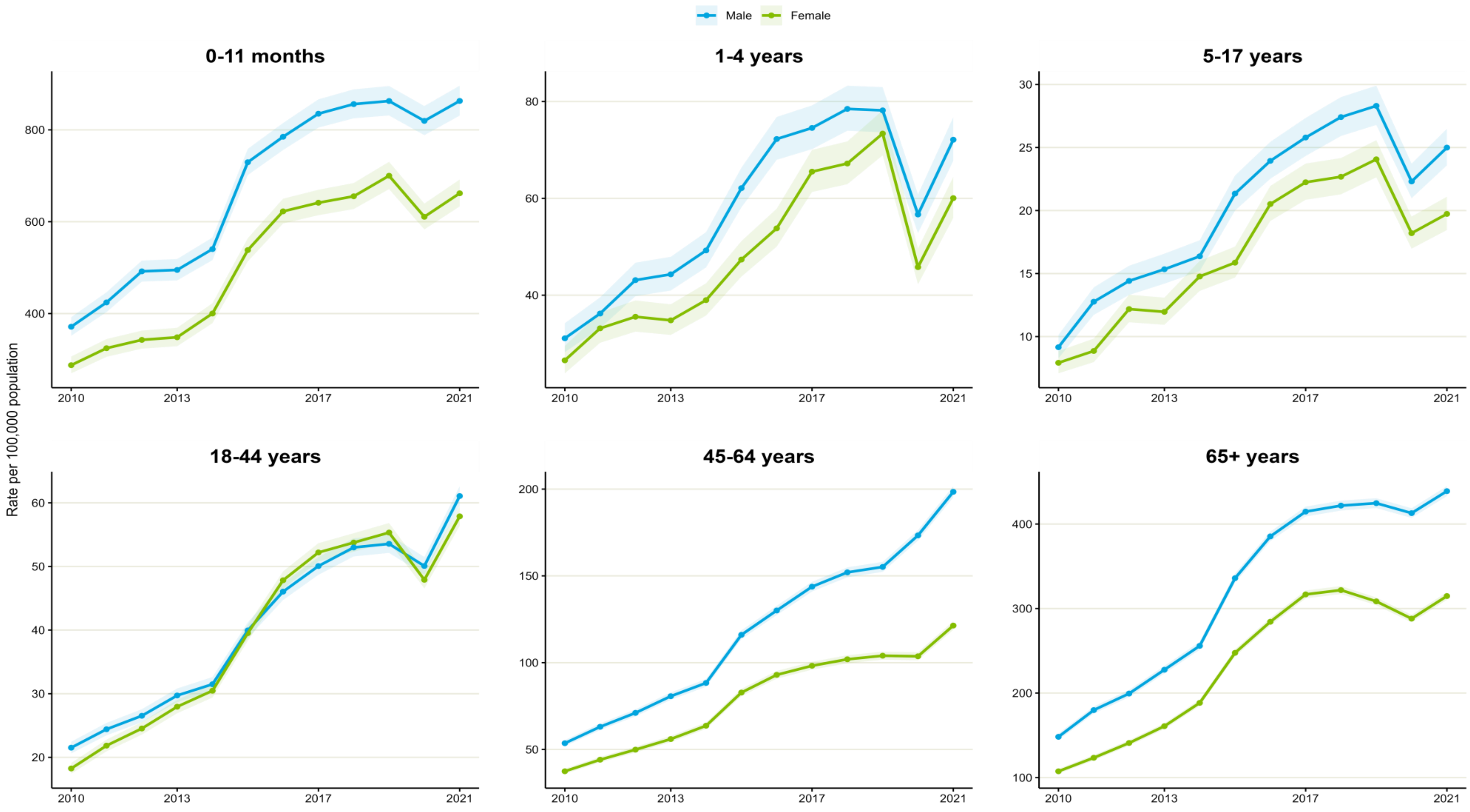

Table S9: Age distribution (in years) of CoNS species

Species	Cases	Median (IQR)
S. epidermidis	174,050	62 (42-75)
S. hominis	43,501	65 (44-77)
S. capitis	25,773	61 (36-76)
S. haemolyticus	17,861	58 (37-71)
S. lugdunensis	6,233	63 (49-75)
S. warneri	6,228	53 (8-71)
S. saccharolyticus	6,219	63 (44-76)
S. cohnii	5,059	61 (40-75)
S. simulans	3,835	67 (56-78)
S. caprae	2,462	65 (50-77)
S. saprophyticus	1,082	56.5 (35-72)
S. pasteuri	878	59 (30-74)
S. auricularis	612	66 (39.8-79)
S. schleiferi	151	75 (62-82)
S. xylosus	128	62 (48.8-75.2)

Table S10: Annual incidence rates (per 100,000) by sex and age group

Age group	Sex	Cases (2010-2021)	Annual incidence rate	Incidence rate ratio	P-value
**0-11 months**	All	46,087	588.3 (582.9-593.6)		
	Female	19,304	505.8 (498.7-513.0)	Reference	<0.001
	Male	26,783	666.6 (658.7-674.6)	1.32 (1.29-1.34)	
**1-4 years**	All	17,342	53.5 (52.8-54.3)		
	Female	7,666	48.5 (47.5-49.6)	Reference	<0.001
	Male	9,676	58.3 (57.2-59.5)	1.20 (1.17-1.24)	
**5-17 years**	All	18,679	18.6 (18.3-18.9)		
	Female	8,197	16.7 (16.4-17.1)	Reference	<0.001
	Male	10,482	20.4 (20.0-20.8)	1.22 (1.19-1.26)	
**18-44 years**	All	94,110	40.2 (39.9-40.5)		
	Female	46,304	39.7 (39.4-40.1)	Reference	<0.001
	Male	47,806	40.7 (40.3-41.0)	1.03 (1.01-1.04)	
**45-64 years**	All	167,464	100.0 (99.5-100.5)		
	Female	68,251	80.5 (79.9-81.1)	Reference	<0.001
	Male	99,213	120.0 (119.3-120.8)	1.49 (1.48-1.51)	
**65+ years**	All	324,167	278.8 (277.8-279.7)		
	Female	151,359	237.9 (236.7-239.1)	Reference	<0.001
	Male	172,808	328.2 (326.7-329.8)	1.38 (1.37-1.39)	
**All ages**	All	667,849	101.4 (101.2-101.7)		
	Female	301,081	90.3 (89.9-90.6)	Reference	<0.001
	Male	366,768	112.9 (112.5-113.2)	1.25 (1.24-1.26)	

Note: Numbers in parenthesis indicate 95% confidence intervals

Figure S3. Number of isolates with susceptibility data

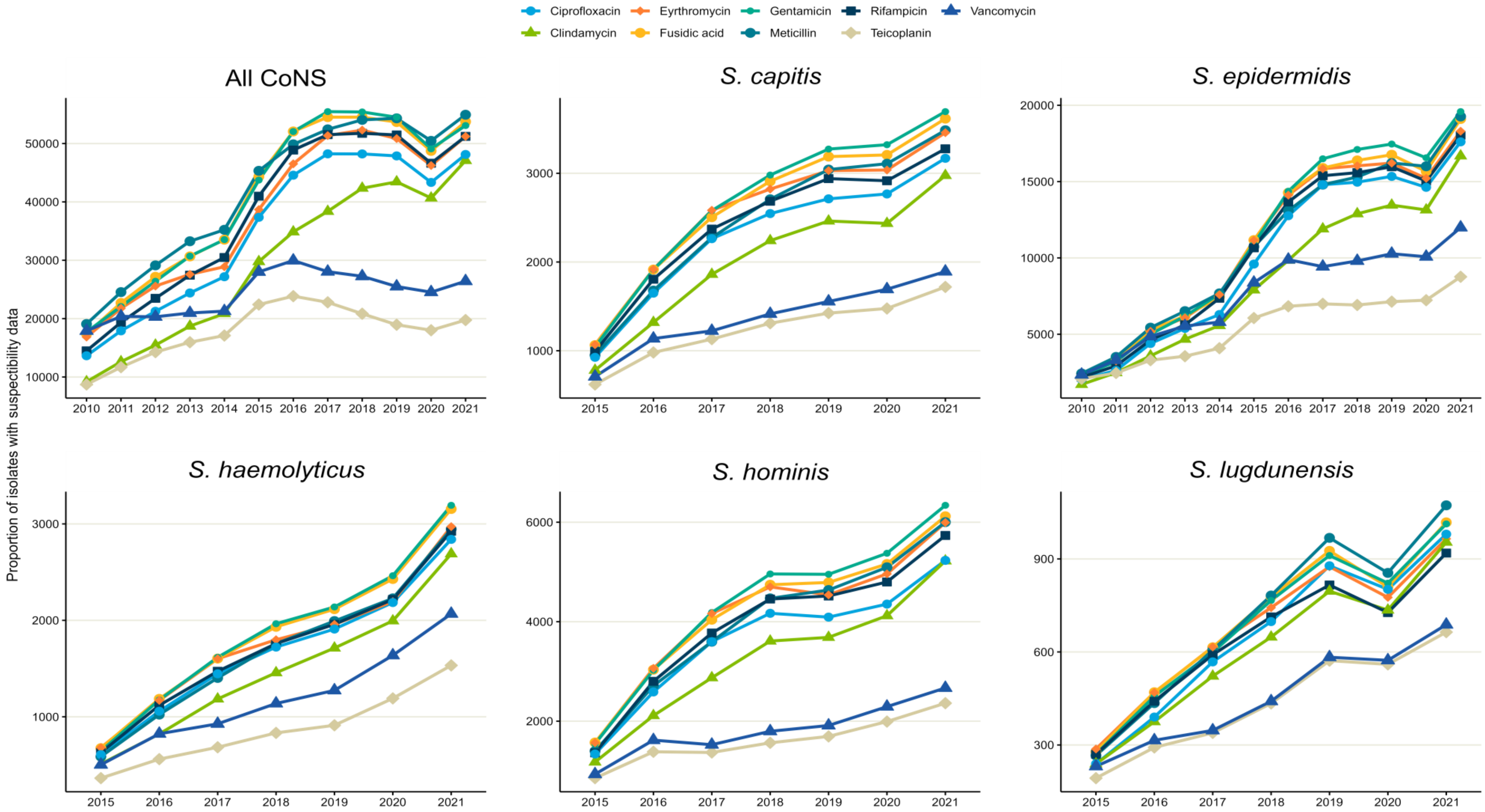

Table S11: Proportion of isolates resistant to antimicrobials - all CoNS

Antimicrobial	2010	2011	2012	2013	2014	2015	2016	2017	2018	2019	2020	2021
Fusidic acid	0.55 (0.54-0.56)	0.56 (0.56-0.57)	0.56 (0.55-0.57)	0.57 (0.56-0.57)	0.58 (0.57-0.58)	0.58 (0.58-0.59)	0.59 (0.58-0.59)	0.59 (0.58-0.59)	0.59 (0.59-0.6)	0.58 (0.58-0.59)	0.6 (0.6-0.61)	0.59 (0.59-0.6)
Meticillin	0.57 (0.57-0.58)	0.57 (0.56-0.58)	0.58 (0.57-0.58)	0.59 (0.59-0.6)	0.6 (0.6-0.61)	0.58 (0.57-0.58)	0.57 (0.56-0.57)	0.55 (0.55-0.56)	0.54 (0.53-0.54)	0.52 (0.51-0.52)	0.52 (0.51-0.52)	0.52 (0.52-0.53)
Gentamicin	0.31 (0.3-0.31)	0.33 (0.32-0.34)	0.34 (0.33-0.34)	0.33 (0.33-0.34)	0.32 (0.32-0.33)	0.32 (0.31-0.32)	0.31 (0.31-0.32)	0.34 (0.34-0.35)	0.35 (0.35-0.36)	0.36 (0.36-0.37)	0.39 (0.39-0.39)	0.41 (0.4-0.41)
Rifampicin	0.11 (0.11-0.12)	0.1 (0.1-0.11)	0.09 (0.09-0.1)	0.08 (0.08-0.09)	0.07 (0.07-0.07)	0.06 (0.06-0.06)	0.05 (0.05-0.06)	0.05 (0.05-0.05)	0.05 (0.05-0.05)	0.05 (0.05-0.05)	0.06 (0.05-0.06)	0.06 (0.06-0.07)
Eyrthromycin	0.6 (0.59-0.61)	0.6 (0.6-0.61)	0.61 (0.61-0.62)	0.63 (0.63-0.64)	0.64 (0.63-0.65)	0.63 (0.63-0.64)	0.63 (0.63-0.64)	0.63 (0.63-0.64)	0.64 (0.63-0.64)	0.63 (0.62-0.63)	0.64 (0.64-0.64)	0.64 (0.63-0.64)
Clindamycin	0.36 (0.35-0.36)	0.37 (0.36-0.37)	0.37 (0.36-0.38)	0.38 (0.38-0.39)	0.41 (0.4-0.42)	0.41 (0.4-0.41)	0.42 (0.41-0.42)	0.44 (0.43-0.44)	0.45 (0.44-0.45)	0.44 (0.43-0.44)	0.46 (0.46-0.47)	0.46 (0.46-0.47)
Ciprofloxacin	0.39 (0.38-0.4)	0.39 (0.39-0.4)	0.39 (0.38-0.4)	0.39 (0.39-0.4)	0.4 (0.39-0.4)	0.37 (0.36-0.37)	0.35 (0.35-0.36)	0.35 (0.35-0.36)	0.35 (0.35-0.36)	0.35 (0.35-0.36)	0.38 (0.38-0.39)	0.39 (0.39-0.39)
Teicoplanin	0.09 (0.09-0.1)	0.13 (0.13-0.14)	0.15 (0.14-0.16)	0.14 (0.13-0.14)	0.14 (0.13-0.14)	0.14 (0.14-0.14)	0.12 (0.12-0.13)	0.13 (0.12-0.13)	0.13 (0.13-0.14)	0.13 (0.12-0.13)	0.13 (0.13-0.14)	0.15 (0.15-0.16)
Vancomycin	0 (0-0.01)	0.01 (0.01-0.01)	0.01 (0.01-0.01)	0.01 (0-0.01)	0.01 (0-0.01)	0.01 (0-0.01)	0 (0-0.01)	0.01 (0-0.01)	0.01 (0-0.01)	0.01 (0.01-0.01)	0.01 (0.01-0.01)	0.01 (0.01-0.01)

Note: Numbers in parenthesis indicate 95% confidence intervals

Table S12: Proportion of isolates resistant to antimicrobials - *S. epidermidis*

Antimicrobial	2010	2011	2012	2013	2014	2015	2016	2017	2018	2019	2020	2021
**Fusidic acid**	0.53 (0.51-0.55)	0.56 (0.55-0.58)	0.59 (0.57-0.6)	0.59 (0.58-0.6)	0.62 (0.61-0.63)	0.64 (0.63-0.65)	0.65 (0.64-0.66)	0.65 (0.64-0.66)	0.66 (0.65-0.67)	0.65 (0.64-0.65)	0.66 (0.65-0.67)	0.64 (0.63-0.65)
**Meticillin**	0.57 (0.55-0.59)	0.6 (0.59-0.62)	0.63 (0.62-0.64)	0.65 (0.64-0.66)	0.67 (0.66-0.68)	0.66 (0.65-0.67)	0.65 (0.64-0.65)	0.66 (0.65-0.67)	0.65 (0.64-0.66)	0.63 (0.62-0.64)	0.64 (0.63-0.65)	0.65 (0.64-0.65)
**Gentamicin**	0.38 (0.36-0.4)	0.41 (0.39-0.42)	0.44 (0.43-0.45)	0.47 (0.45-0.48)	0.46 (0.45-0.47)	0.45 (0.44-0.46)	0.43 (0.43-0.44)	0.48 (0.47-0.48)	0.49 (0.48-0.49)	0.5 (0.49-0.51)	0.53 (0.52-0.54)	0.55 (0.54-0.56)
**Rifampicin**	0.13 (0.12-0.14)	0.14 (0.13-0.16)	0.14 (0.13-0.15)	0.13 (0.12-0.14)	0.1 (0.09-0.11)	0.1 (0.09-0.1)	0.09 (0.09-0.1)	0.08 (0.07-0.08)	0.08 (0.07-0.08)	0.07 (0.07-0.08)	0.08 (0.07-0.08)	0.07 (0.07-0.08)
**Eyrthromycin**	0.63 (0.61-0.65)	0.63 (0.62-0.65)	0.66 (0.65-0.68)	0.69 (0.68-0.7)	0.71 (0.7-0.72)	0.7 (0.69-0.71)	0.71 (0.7-0.72)	0.71 (0.7-0.72)	0.72 (0.71-0.72)	0.71 (0.7-0.72)	0.73 (0.72-0.73)	0.72 (0.71-0.72)
**Clindamycin**	0.35 (0.32-0.37)	0.4 (0.38-0.42)	0.42 (0.4-0.44)	0.46 (0.45-0.48)	0.5 (0.49-0.52)	0.51 (0.5-0.52)	0.52 (0.51-0.53)	0.54 (0.53-0.55)	0.54 (0.54-0.55)	0.54 (0.53-0.54)	0.56 (0.55-0.57)	0.56 (0.55-0.56)
**Ciprofloxacin**	0.44 (0.42-0.46)	0.44 (0.42-0.46)	0.49 (0.47-0.5)	0.51 (0.49-0.52)	0.53 (0.52-0.54)	0.51 (0.5-0.52)	0.49 (0.48-0.5)	0.49 (0.48-0.5)	0.49 (0.48-0.5)	0.49 (0.49-0.5)	0.53 (0.52-0.54)	0.54 (0.53-0.54)
**Teicoplanin**	0.1 (0.08-0.11)	0.15 (0.14-0.17)	0.19 (0.18-0.2)	0.22 (0.21-0.24)	0.21 (0.19-0.22)	0.2 (0.19-0.21)	0.19 (0.18-0.2)	0.2 (0.19-0.21)	0.18 (0.17-0.19)	0.15 (0.15-0.16)	0.17 (0.16-0.18)	0.19 (0.18-0.2)
**Vancomycin**	0.01 (0.01-0.02)	0.01 (0.01-0.02)	0.02 (0.02-0.02)	0.01 (0.01-0.02)	0.01 (0.01-0.01)	0.01 (0.01-0.01)	0.01 (0.01-0.01)	0.01 (0.01-0.01)	0.01 (0.01-0.01)	0.01 (0.01-0.01)	0.01 (0.01-0.01)	0.01 (0.01-0.01)

Note: Numbers in parenthesis indicate 95% confidence intervals

Table S13: Proportion of isolates resistant to antimicrobials - *S. capitis*

Antimicrobial	2015	2016	2017	2018	2019	2020	2021
Fusidic acid	0.38 (0.35-0.41)	0.38 (0.36-0.4)	0.41 (0.39-0.43)	0.44 (0.42-0.45)	0.43 (0.41-0.45)	0.44 (0.43-0.46)	0.46 (0.45-0.48)
Meticillin	0.43 (0.4-0.46)	0.41 (0.38-0.43)	0.35 (0.33-0.37)	0.34 (0.32-0.36)	0.33 (0.32-0.35)	0.34 (0.32-0.36)	0.32 (0.31-0.34)
Gentamicin	0.22 (0.2-0.25)	0.23 (0.22-0.25)	0.22 (0.21-0.24)	0.24 (0.22-0.25)	0.25 (0.24-0.27)	0.26 (0.25-0.28)	0.25 (0.24-0.27)
Rifampicin	0.02 (0.01-0.02)	0.01 (0.01-0.02)	0.01 (0.01-0.01)	0.01 (0.01-0.02)	0.01 (0.01-0.01)	0.01 (0.01-0.02)	0.01 (0.01-0.02)
Eyrthromycin	0.28 (0.25-0.31)	0.3 (0.28-0.32)	0.28 (0.26-0.3)	0.3 (0.29-0.32)	0.3 (0.29-0.32)	0.31 (0.29-0.32)	0.29 (0.28-0.31)
Clindamycin	0.18 (0.15-0.21)	0.18 (0.16-0.2)	0.18 (0.17-0.2)	0.2 (0.18-0.22)	0.18 (0.16-0.19)	0.2 (0.18-0.22)	0.2 (0.19-0.21)
Ciprofloxacin	0.11 (0.09-0.13)	0.12 (0.11-0.14)	0.13 (0.12-0.15)	0.14 (0.13-0.16)	0.15 (0.14-0.16)	0.17 (0.16-0.19)	0.19 (0.17-0.2)
Teicoplanin	0.11 (0.09-0.14)	0.1 (0.08-0.12)	0.12 (0.1-0.14)	0.13 (0.11-0.15)	0.12 (0.1-0.13)	0.11 (0.09-0.12)	0.1 (0.09-0.12)
Vancomycin	0 (0-0.01)	0 (0-0.01)	0 (0-0)	0 (0-0.01)	0 (0-0.01)	0.01 (0-0.01)	0.01 (0-0.01)

Note: Numbers in parenthesis indicate 95% confidence intervals

Table S14: Proportion of isolates resistant to antimicrobials - *S. haemolyticus*

Antimicrobial	2015	2016	2017	2018	2019	2020	2021
Fusidic acid	0.38 (0.34-0.42)	0.36 (0.33-0.39)	0.41 (0.39-0.43)	0.43 (0.41-0.45)	0.47 (0.45-0.5)	0.47 (0.45-0.49)	0.45 (0.43-0.46)
Meticillin	0.81 (0.78-0.84)	0.77 (0.74-0.79)	0.8 (0.78-0.82)	0.78 (0.76-0.8)	0.79 (0.77-0.81)	0.83 (0.81-0.84)	0.83 (0.82-0.84)
Gentamicin	0.66 (0.62-0.69)	0.66 (0.63-0.68)	0.68 (0.65-0.7)	0.68 (0.66-0.7)	0.7 (0.68-0.71)	0.74 (0.73-0.76)	0.76 (0.74-0.77)
Rifampicin	0.1 (0.08-0.12)	0.1 (0.08-0.12)	0.13 (0.11-0.15)	0.15 (0.14-0.17)	0.17 (0.15-0.19)	0.24 (0.22-0.26)	0.31 (0.3-0.33)
Eyrthromycin	0.87 (0.84-0.89)	0.81 (0.79-0.84)	0.84 (0.82-0.85)	0.84 (0.83-0.86)	0.85 (0.83-0.86)	0.86 (0.85-0.88)	0.85 (0.84-0.86)
Clindamycin	0.51 (0.47-0.56)	0.56 (0.53-0.6)	0.59 (0.56-0.62)	0.6 (0.58-0.63)	0.59 (0.57-0.61)	0.66 (0.64-0.68)	0.66 (0.64-0.68)
Ciprofloxacin	0.59 (0.55-0.62)	0.6 (0.57-0.63)	0.63 (0.61-0.66)	0.65 (0.63-0.67)	0.65 (0.63-0.67)	0.7 (0.68-0.72)	0.7 (0.69-0.72)
Teicoplanin	0.09 (0.07-0.12)	0.13 (0.1-0.16)	0.08 (0.06-0.11)	0.12 (0.1-0.14)	0.13 (0.11-0.15)	0.13 (0.11-0.15)	0.17 (0.15-0.19)
Vancomycin	0 (0-0.01)	0 (0-0.01)	0 (0-0.01)	0 (0-0)	0 (0-0.01)	0.01 (0.01-0.02)	0.01 (0-0.01)

Note: Numbers in parenthesis indicate 95% confidence intervals

Table S15: Proportion of isolates resistant to antimicrobials - *S. hominis*

Antimicrobial	2015	2016	2017	2018	2019	2020	2021
Fusidic acid	0.68 (0.66-0.7)	0.7 (0.68-0.71)	0.69 (0.68-0.71)	0.7 (0.68-0.71)	0.69 (0.68-0.71)	0.7 (0.69-0.72)	0.68 (0.67-0.69)
Meticillin	0.52 (0.49-0.55)	0.51 (0.49-0.53)	0.49 (0.48-0.51)	0.46 (0.45-0.48)	0.44 (0.43-0.46)	0.43 (0.42-0.44)	0.42 (0.4-0.43)
Gentamicin	0.15 (0.13-0.16)	0.13 (0.12-0.14)	0.16 (0.15-0.17)	0.17 (0.16-0.18)	0.19 (0.18-0.2)	0.2 (0.19-0.21)	0.2 (0.19-0.21)
Rifampicin	0.01 (0.01-0.02)	0.01 (0.01-0.01)	0.01 (0.01-0.01)	0.01 (0.01-0.02)	0.01 (0.01-0.01)	0.01 (0.01-0.02)	0.01 (0.01-0.02)
Eyrthromycin	0.64 (0.62-0.66)	0.64 (0.62-0.66)	0.66 (0.65-0.68)	0.65 (0.64-0.66)	0.66 (0.64-0.67)	0.65 (0.64-0.66)	0.64 (0.63-0.66)
Clindamycin	0.36 (0.33-0.39)	0.36 (0.34-0.38)	0.38 (0.36-0.4)	0.39 (0.37-0.4)	0.38 (0.36-0.39)	0.38 (0.36-0.39)	0.38 (0.37-0.4)
Ciprofloxacin	0.16 (0.14-0.18)	0.14 (0.12-0.15)	0.15 (0.14-0.16)	0.16 (0.15-0.17)	0.16 (0.15-0.17)	0.17 (0.16-0.18)	0.16 (0.15-0.17)
Teicoplanin	0.06 (0.04-0.07)	0.06 (0.05-0.07)	0.04 (0.03-0.05)	0.06 (0.05-0.07)	0.06 (0.05-0.08)	0.05 (0.04-0.06)	0.06 (0.06-0.07)
Vancomycin	0 (0-0.01)	0 (0-0)	0 (0-0.01)	0 (0-0)	0 (0-0.01)	0 (0-0.01)	0 (0-0.01)

Note: Numbers in parenthesis indicate 95% confidence intervals

Table S16: Proportion of isolates resistant to antimicrobials - *S. lugdunensis*

Antimicrobial	2015	2016	2017	2018	2019	2020	2021
Fusidic acid	0.09 (0.06-0.13)	0.11 (0.08-0.14)	0.12 (0.09-0.14)	0.11 (0.09-0.13)	0.1 (0.09-0.13)	0.09 (0.08-0.12)	0.12 (0.1-0.15)
Meticillin	0.14 (0.1-0.18)	0.07 (0.05-0.1)	0.07 (0.05-0.1)	0.07 (0.05-0.09)	0.06 (0.05-0.08)	0.06 (0.05-0.08)	0.08 (0.06-0.1)
Gentamicin	0.04 (0.02-0.07)	0.03 (0.02-0.05)	0.03 (0.02-0.04)	0.03 (0.02-0.04)	0.03 (0.02-0.04)	0.03 (0.02-0.04)	0.03 (0.02-0.04)
Rifampicin	0.03 (0.01-0.05)	0.01 (0-0.02)	0.02 (0.01-0.03)	0.01 (0-0.02)	0.01 (0-0.02)	0.01 (0.01-0.02)	0.01 (0-0.01)
Eyrthromycin	0.13 (0.09-0.17)	0.1 (0.08-0.14)	0.12 (0.09-0.14)	0.14 (0.12-0.17)	0.12 (0.1-0.15)	0.12 (0.1-0.15)	0.13 (0.11-0.15)
Clindamycin	0.13 (0.1-0.18)	0.12 (0.09-0.15)	0.12 (0.09-0.15)	0.14 (0.12-0.17)	0.13 (0.11-0.16)	0.11 (0.09-0.14)	0.12 (0.1-0.14)
Ciprofloxacin	0.03 (0.01-0.05)	0.02 (0.01-0.04)	0.03 (0.02-0.04)	0.03 (0.02-0.05)	0.03 (0.02-0.05)	0.04 (0.03-0.06)	0.03 (0.02-0.05)
Teicoplanin	0.01 (0-0.03)	0 (0-0.02)	0.01 (0-0.03)	0.02 (0.01-0.03)	0.01 (0-0.02)	0 (0-0.01)	0 (0-0.01)
Vancomycin	0 (0-0.02)	0 (0-0.02)	0 (0-0.01)	0 (0-0.01)	0 (0-0.01)	0 (0-0.01)	0 (0-0.01)

Note: Numbers in parenthesis indicate 95% confidence intervals

Figure S4. Number of laboratories reporting yearly by CoNS species

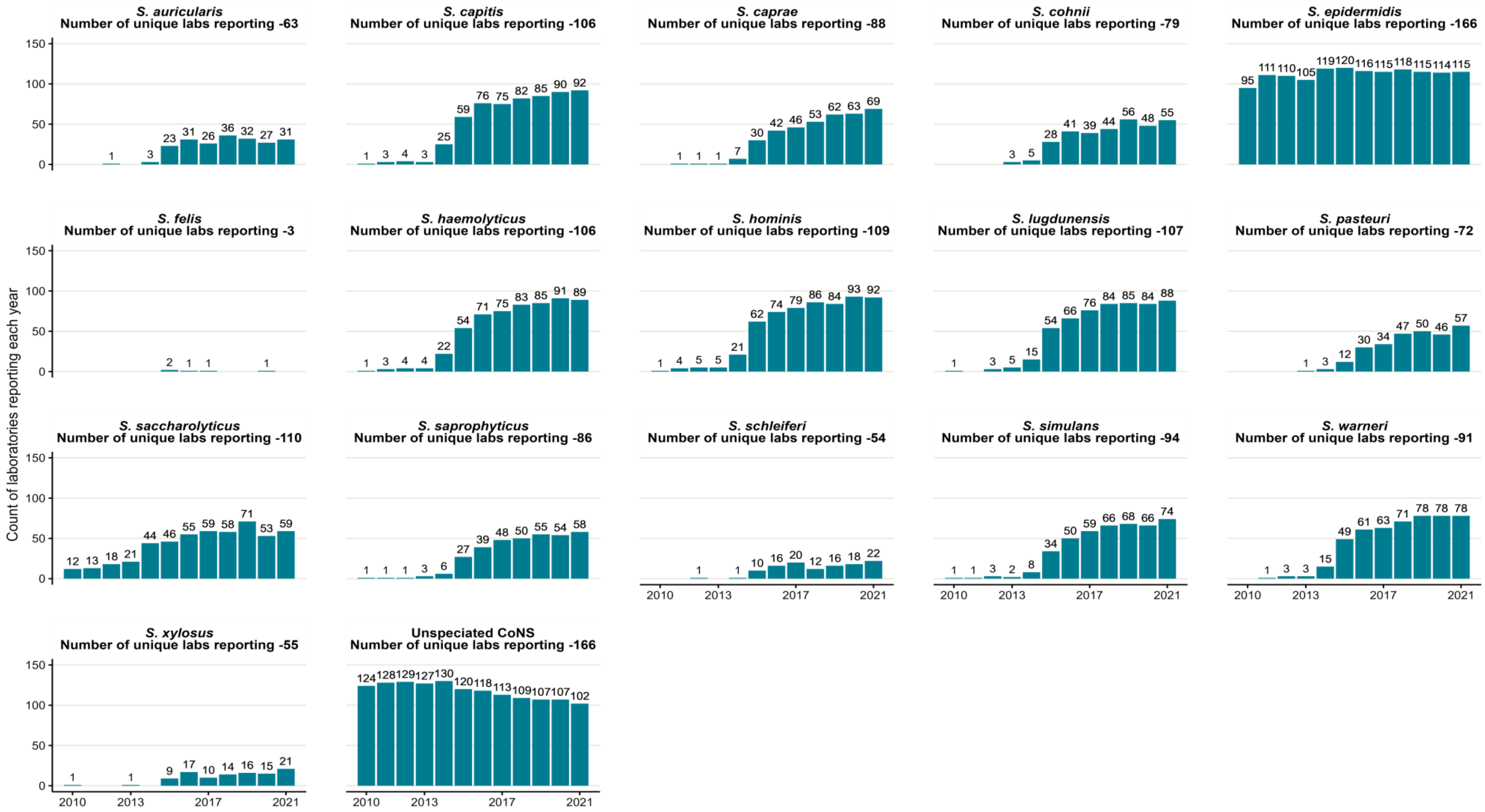

Figure S5. Number of unique species reported by laboratories by year

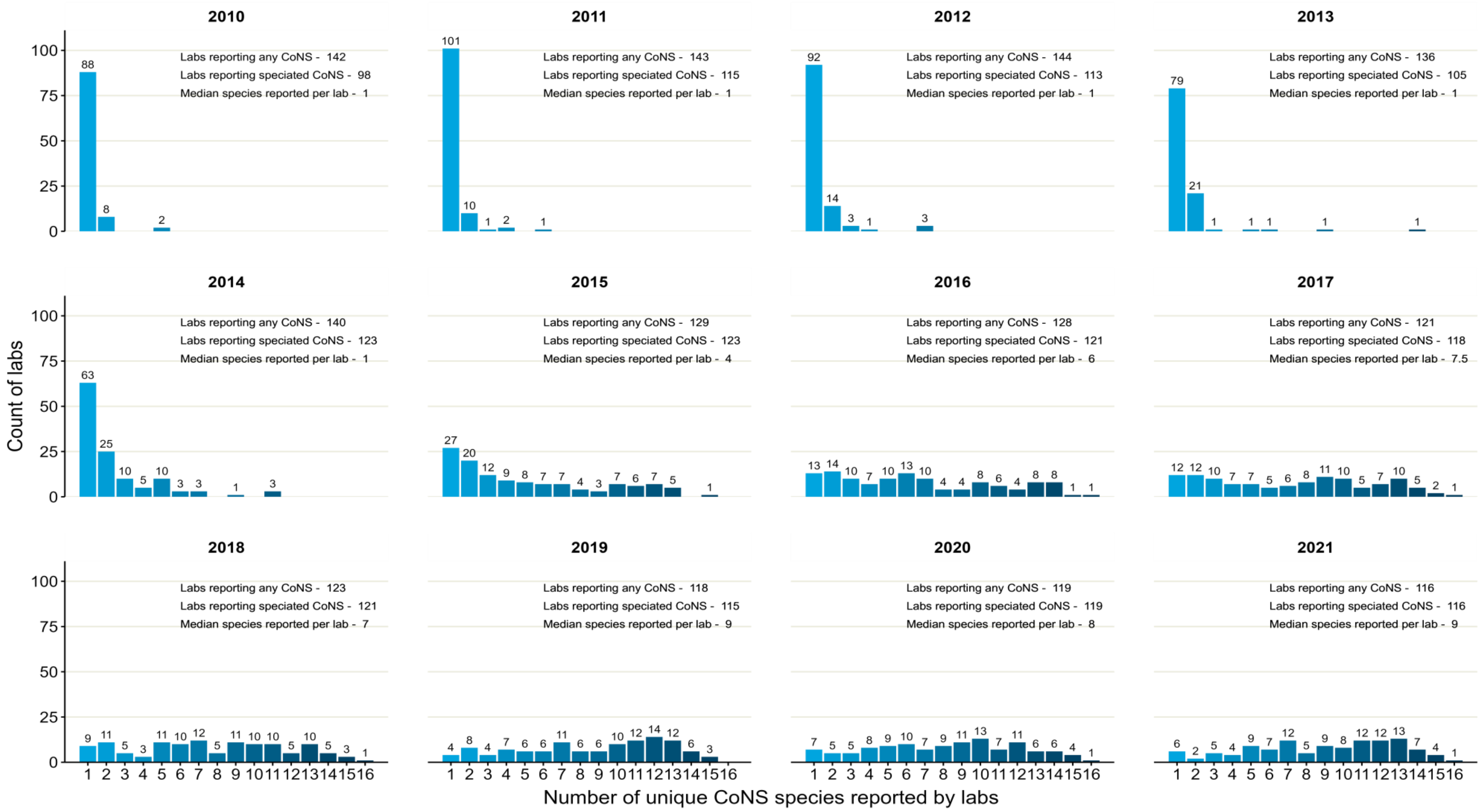

Supplement: Supplementary material 1 [file acmi-5-491.v3-s001.pdf]
